# Utilization and Perceived Problems of Online Medical Resources and Search Tools Among Different Groups of European Physicians

**DOI:** 10.2196/jmir.2436

**Published:** 2013-06-26

**Authors:** Marlene Kritz, Manfred Gschwandtner, Veronika Stefanov, Allan Hanbury, Matthias Samwald

**Affiliations:** ^1^Society of Physicians ViennaViennaAustria; ^2^Information & Software Engineering GroupInstitute of Software Technology and Interactive SystemsVienna University of TechnologyViennaAustria; ^3^Section for Medical Expert and Knowledge-Based SystemsCenter for Medical Statistics, Informatics, and Intelligent SystemsMedical University of ViennaViennaAustria

**Keywords:** information seeking behavior, physicians, Internet, search engine, information quality, language barriers

## Abstract

**Background:**

There is a large body of research suggesting that medical professionals have unmet information needs during their daily routines.

**Objective:**

To investigate which online resources and tools different groups of European physicians use to gather medical information and to identify barriers that prevent the successful retrieval of medical information from the Internet.

**Methods:**

A detailed Web-based questionnaire was sent out to approximately 15,000 physicians across Europe and disseminated through partner websites. 500 European physicians of different levels of academic qualification and medical specialization were included in the analysis. Self-reported frequency of use of different types of online resources, perceived importance of search tools, and perceived search barriers were measured. Comparisons were made across different levels of qualification (qualified physicians vs physicians in training, medical specialists without professorships vs medical professors) and specialization (general practitioners vs specialists).

**Results:**

Most participants were Internet-savvy, came from Austria (43%, 190/440) and Switzerland (31%, 137/440), were above 50 years old (56%, 239/430), stated high levels of medical work experience, had regular patient contact and were employed in nonacademic health care settings (41%, 177/432). All groups reported frequent use of general search engines and cited “restricted accessibility to good quality information” as a dominant barrier to finding medical information on the Internet. Physicians in training reported the most frequent use of Wikipedia (56%, 31/55). Specialists were more likely than general practitioners to use medical research databases (68%, 185/274 vs 27%, 24/88; χ^2^_2_=44.905, *P*<.001). General practitioners were more likely than specialists to report “lack of time” as a barrier towards finding information on the Internet (59%, 50/85 vs 43%, 111/260; χ^2^_1_=7.231, *P*=.007) and to restrict their search by language (48%, 43/89 vs 35%, 97/278; χ^2^_1_=5.148, *P*=.023*). They* frequently consult general health websites (36%, 31/87 vs 19%, 51/269; χ^2^_2_=12.813, *P*=.002) and online physician network communities (17%, 15/86, χ^2^_2_=9.841 vs 6%, 17/270, *P*<.001).

**Conclusions:**

The reported inaccessibility of relevant, trustworthy resources on the Internet and frequent reliance on general search engines and social media among physicians require further attention. Possible solutions may be increased governmental support for the development and popularization of user-tailored medical search tools and open access to high-quality content for physicians. The potential role of collaborative tools in providing the psychological support and affirmation normally given by medical colleagues needs further consideration. Tools that speed up quality evaluation and aid selection of relevant search results need to be identified. In order to develop an adequate search tool, a differentiated approach considering the differing needs of physician subgroups may be beneficial.

##  Introduction

### Background

Over the last decades, the World Wide Web has become an important source of information within the medical health domain [1]. Physicians’ information needs, information-seeking behavior, and their use of online resources have been well studied [2]. Physicians primarily access the Internet at the point of care [3] to pursue medical updating and to communicate with colleagues [4]. The Internet gives medical professionals access to a vast amount of high-quality medical information, which could potentially aid medical decision making and patient care. It has been found that 51% of physicians claim that the Web has influenced treatment and assisted them in diagnostic procedures [5], and 30% of a sample of 411 physicians declared “often” changing medication and treatment plans as a consequence of obtaining information from the Internet [6]. The benefit and usability of medical information provided on the Internet increasingly relies on adequate content, quality evaluation, and the skilled selection of relevant websites. To improve on how computers can help physicians with medical information retrieval, an understanding of online resource and search requirements of physicians in different health care settings is required.

### Use of Resources

There has been some research discussing what makes a source useful for medical professionals. Shaughnessy and colleagues [7] have measured the usefulness of information resources with the formula: 

Utility=(relevance x validity x interactivity)/work to access

According to the formula, the ideal information source is directly relevant, contains valid information, and can be accessed with a minimal amount of work. Sources with low “work to access” (ie, easy accessibility) and high surface relevance—such as general search engines and colleagues—are often reported as popular among time-constrained medical professionals [3,8]. However, answers provided by such sources often lack scientific validity. In contrast, medical research databases such as PubMed can provide journal articles and systematic reviews backed up by good evidence but often fail to quickly provide relevant content, resulting in a considerable amount of mental “work to access” [9]. In addition, point-of-care databases such as UpToDate are associated with high levels of economic “work to access” as they provide relevant and valid information but require the payment of substantial subscription fees to be accessible. Consequently, “low work to access” resources such as general search engines and colleagues have remained dominant information resources within the medical domain [6]. The role of the “psychological support and affirmation” provided by a medical colleague has remained dominant and irreplaceable [10]. However, while it has been postulated that general search engines such as Google can aid the diagnostic process [8] and can be efficient at answering quick questions within a critical care setting [9], it is questionable whether physicians always have the time and expertise to pursue the necessary data selection.

### Barriers

It has been well documented that physicians face barriers that keep them from finding relevant information to their queries [11]. However, to date, there is a lack of research treating physicians as a heterogeneous group and comparing potential search barriers in different subgroups of physicians. A differentiated understanding of problems associated with current search systems could be beneficial towards the development of efficient user-tailored medical search systems.

### Tools

Tools to overcome search barriers by simplifying content selection and quality evaluation of information are of growing interest within the medical search domain. There is a lack of knowledge of the tools physicians prefer to use when searching for online information. In particular, there is a lack of data on the acceptance of social, collaborative tools for aiding the selection of relevant search results.

### Objective

This paper aims to provide insight on the professional Internet use among different subgroups of physicians: their use of resources, tools, and potential barriers in obtaining answers from the Internet. We are expanding current knowledge in this area by presenting a large quantitative survey among physicians located in several European countries and employed in a wide variety of health care settings. To date, no detailed, large-scale quantitative study has been published on the use of online resources, preferences of search tools, and search barriers of different subgroups of European physicians. Most studies in the field have been carried out in countries where English is the primary language of communication. Thus, the role of nonEnglish languages in online search behavior of medical professionals has remained unclear.

Our main objective was to analyze the use of online resources, search tools, and perceived search barriers among European physicians. We compared different subgroups of physicians to determine whether level of academic qualification, type of medical specialization, and medical experience could impact search behavior.

The outcomes of the present survey could assist the future development of effective medical search systems and provide orientation for the potential creation of institutional policies on using the Web in health care settings.

## Methods

This survey is part of the project KHRESMOI, funded by the European Union. One of the goals of the project is to investigate the search requirements of physicians in order to guide the development of a medical search engine.

### Questionnaire

An online questionnaire based on categorical assumptions of previous qualitative literature, on medical information needs [12], use of online resources [13], and physician search behavior and preferences [6] was developed. While previous research provided a good starting point for the questionnaire design, most items were modified, extended, or deleted to fit the requirements of European physicians after doing preliminary, structured pilot interviews with Austrian physicians and disseminating a pilot questionnaire to Austrian, Spanish, Swiss, and English physicians. We used informal semistructured interviews to develop the questionnaire. The questionnaire was validated via an “online test period” of 1 week, in which 12 physicians completed the survey and provided subsequent feedback.

The final version of the survey questionnaire (see Multimedia Appendix 1) consisted of 6 parts encompassing a total of 47 questions and was made available online in English, German, French, and Spanish on an open-access platform hosted by Health on the Net [14]. The questionnaire could be accessed by clicking on a link that was included in the emails sent out, or by clicking on a banner on the Health on the Net website. The number of items displayed per page varied from 1 to 14, and it was ensured that each part was displayed on one page. The whole questionnaire was spread over a total of 8 pages. All items contained the option of a nonresponse item (eg, “not applicable”) and/or an open-format “other item”. Participants were allowed to change responses using the back button. Computer Internet Protocol (IP) addresses were tracked and used to identify duplicate entries. In the case of duplicate entries within a time frame of 12 hours, the first entry was excluded from the analysis. The study was anonymous. Each participant was presented with an information sheet and asked to confirm being a physician/medical student prior to completing the questionnaire. The questionnaire was edited in simple HTML without Java applets or special scripts to allow a maximum number of the addressed users to access it with their browsers.

Most questions were either of multiple response/dichotomous questions or could be answered by selecting an option on a 5-point Likert scale rating. In our analysis, we included 15 sociodemographic variables and present the results of 14 selected items related to the use of resources, tools, and barriers (Table 1).

**Table 1:** Subgroups of physicians.

	Definition/inclusion criteria
Physician in training	Physicians that had completed a medical degree (MD) but were currently pursuing physician training to become a general practitioner or specialist.
Qualified physicians	Included all physicians that were working as qualified physicians (general practitioners and specialists)
General practitioners	Included all physicians that were working as qualified general practitioners
Specialists	Included all physicians who are were working as qualified specialists
Specialists without professorship	A subgroup of the specialists group, included physicians who are qualified specialists without having attained lectureship or professorship
Medical professors	A subgroup of the specialists group, included physicians who had completed a postgraduate lectureship qualification or were appointed university professors

### Study Population

The target population consisted of European physicians of all specialities and was a convenience sample. The questionnaire was promoted in June and July 2011 by email at random to about 15,000 physicians around Europe through a banner on websites, medical newsletters, and an article in an Austrian public health journal with approximately 35,000 readers. The following institutions sent out newsletters with a link and information on the questionnaire: Society of Physicians Vienna (2800 members), Geneva Doctors Association (3100 members), Austrian Society of Internal Medicine (2100 members), Austrian Society for Gastroenterology and Hepatology (800 members), and Medical Media in Austria (5500 members). The following institutions placed a banner pointing to the questionnaire on their websites: Doctors.net.uk, Society of Physicians in Vienna, Health on the Net Foundation, Professional Association of German Internists, and the European Academy of Allergy and Clinical Immunology.

### Statistical Analysis

Statistical analyses were performed using SPSS 21 for Windows. Tables and graphs were constructed using Microsoft Office Excel 2007. Due to unsolved controversies in the literature of reliably treating Likert-items as interval-scaled, 5-point Likert items were treated as ordinal and other items as nominal or dichotomized [15]. Thus a nonparametric approach, believed to be the most reliable method for such data [16], was used for all statistical inferences.

Differences between independent variables—medical specialization (specialist vs general practitioner), level of qualification (physician in training vs qualified physician), and academic qualification (medical professor vs medical specialist without professorship)—were initially explored using a Mann-Whitney U Test for all 5-point Likert scale items. However, since the distributions of most of the ordinal 5-point Likert-items were skewed, we decided to simplify all responses on 5-point Likert scales by re-categorizing responses into only three items (“always/often”, “sometimes”, “rarely/never”, or similarly “very important/important”, “neutral”, “unimportant”). Multiple response questions were coded and analyzed as dichotomous yes/no variables.

The questionnaire examined various sociodemographic variables, the use of online resources, available time, search strategy, barriers, search tools used, advanced search features used, and mobile accessibility. The sample population was split into subgroups of physicians, as defined in Table 1.

Questionnaire results were analyzed to provide descriptive statistics, graphs, and cross-tabulations. A Chi-square analysis was performed to assess differences between ordinal dependent variables with two to four categories as well as all dichotomous and nominal data. A *P* value ≤.05 was considered as statistically significant.

### Exclusion Criteria

Participants were excluded if they answered fewer than 30% of the questions (ie, at least 14 of the 46 questions had to be answered), did not complete their medical degree yet, or were not involved in patient treatment. The low threshold of inclusion was chosen to deliberately allow for partially filled out questionnaires. For example, it was important to include physicians who selectively failed to fill out the demographics section (about 23% of the total sample included in the analysis), to avoid creating a bias by excluding physicians who merely wanted to assure additional confidentiality.

## Results

Of the initial 640 participants taking part in the study, 140 were excluded based on the selection criteria described earlier. This resulted in a sample of 500 participants that had completed all or a substantial part of the questionnaire being included in the analysis. Results are reported as a percentage of the total number of responses to each question (the denominator therefore varies according to the individual question response rate). No statistical corrections such as weighting were used. However, nonresponders to individual questions were excluded from the analysis of those questions.

### Demographics

In total, 63% (271/430) of the participants were male, and 56% (239/430) of the participants were older than 50 years. Most participants came from the main areas of questionnaire dissemination: the majority came from Austria (43%, 190/440), almost a third from Switzerland (31%, 137/440), followed by the United Kingdom (8%, 37/440), and Germany (4%, 17/440). Respectively, most participants spoke German (46%, 198/429) or French (31%, 133/429). The majority of the participants (81%, 345/427) reported living in urban areas. The reported level of education and medical work experience was exceptionally high; 13% of the physicians (54/432) reported having completed medical lectureship training. More than half of the respondents reported having obtained work experience of “20 years or more” (55%, 238/428). In fact, 96% (415/432) reported currently working as medical professionals. Most were self-employed (39%, 168/432) or worked in nonacademic health care settings (41%, 177/432), only 4% (17/432) identified as retired or unemployed, and 64% (278/432) of the physicians identified as working specialists, 21% (89/432) as general practitioners, and 13% (55/432) reported pursuing physician training. 90% (387/429) of the physicians reported seeing patients on a regular basis. However, the majority (87%, 337/387) of those with regular patient contact consulted less than 40 patients per working day.

Overall, levels of self-perceived competence of medical English were high. Among the nonnative English speakers (91%, 391/429), 89% (348/391) of the physicians reported that their level of medical English is above average, with only 1 participant reporting not understanding any English. While 99% (492/498) reported having regular Internet access, 92% (454/496) reported using the Internet on a daily basis. High levels of Internet experience were reported, with 82% (408/499) using the Internet for more than 10 years. In addition, of those physicians who reported having regular direct patient contact (90%, 387/429), as many as 24% (92/385) reported that they frequently (“often” or “always”) access the Internet during a patient consultation), and 44% (171/385) reported never accessing the Internet during a patient consultation.

Table 2 illustrates how the physicians in the sample were distributed among the subgroups we defined based on medical qualification, medical specialization, and academic qualification.

**Table 2:** Comparisons between different groups of physicians.

	Medical qualification		Medical specialization		Academic qualification	
	Physician in training	Qualified physician	General practitioner	Specialist	Specialist without professorship	Medical professor
n/N^a^	55/422	367/422	89/367	278/367	224/278	54/278
% of N	13	87	24	76	81	19

^a^N is based on the number of respondents that provided a response to the corresponding question in the questionnaire.

###  Use of Online Resources

**Figure 1:**
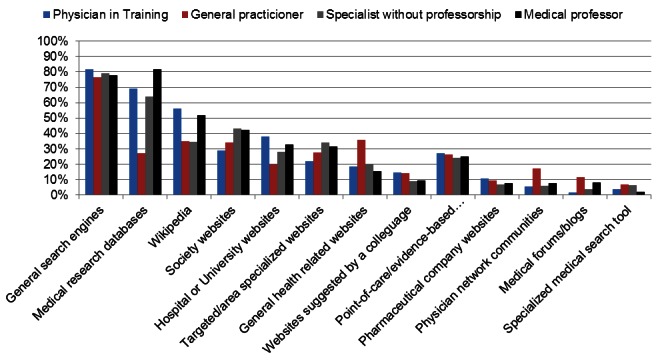
Online resources used for obtaining medical information.

The frequencies with which physicians reported using various types of online resources are shown in Figure 1 and in Table 1 in Multimedia Appendix 2. Figure 1 illustrates the percentage of respondents per group that claimed to use a given resource “often” or “always” when searching for medical information on the Internet. Numerical values and statistical parameters are shown in Table 1 in Multimedia Appendix 2. Most physicians reported frequently using general search engines (78%, 372/476), medical research databases (59%, 277/469), Wikipedia (40%, 184/461), or medical society websites (38%, 176/467) to obtain medical information online. Currently available specialized medical search tools were reported as the least popular resources (6%, 25/458).

Both the level of medical specialization and level of academic experience were associated with how often physicians consulted medical research databases. Medical professors were the only group that cited using medical research databases (82%, 44/54) as often as general search engines (75%, 42/54) during their search for medical information. Specialists reported using medical research databases more frequently than general practitioners (68%, 185/274 vs 27%, 24/88; χ^2^_2_=44.905, *P*<.001). On the other hand, general practitioners were more likely than specialists to consult general health websites (36%, 31/87 vs 19%, 51/269; χ^2^_2_=12.813, *P*=.002), online physician communities (17%, 15/86 vs 6%, 17/270; χ^2^_2_=9.841, *P*<.001), and medical forums/blogs (12%, 10/86 vs 5%, 12/266; χ^2^_2_=9.841, *P*<.01). Medical professors were more likely than specialists without professorship to use medical research databases (82%, 44/54 vs 64%, 141/220; χ^2^_2_=7.461, *P*=.024), and Wikipedia (52%, 27/52 vs 34%, 74/215; χ^2^_2_=7.461, *P*=.024). Physicians in training were more likely to report using Wikipedia (56%, 31/55 vs 37%, 131/353; χ^2^_2_=8.997, *P*=.011), hospital/university websites (38%, 21/55 vs 27%, 95/357; χ^2^_2_=6.409, *P*=.041), and websites suggested by colleagues (15%, 8/54 vs 10%, 37/355; χ^2^_2_=8.653, *P*=.001) than qualified physicians. Qualified physicians were more likely than physicians in training to consult general medical society websites (41%, 147/361 vs 29%, 16/55; χ^2^_2_=11,622, *P*=.003).

Overall, online physician communities were relatively unknown; 28% (116/419) of the total sample was unaware of their existence. Only 24% of the total sample (72/303) reported using such communities. Of those who did use them, the most popular physician communities reported were “Doctors.net” (n=10) and “doc2doc” (n=8). However, 33% of general practitioners (29/89) and 24% (65/276) of the specialists were unaware of the existence of physician society communities. However, among the physicians who were aware of their existence, general practitioners were more likely to access physician society community websites than specialists (χ^2^_1_=19.083, *P*<.001).

We asked physicians which restriction criteria they used during their search for medical information. Overall, journals (48%, 150/333), source (35%, 136/391), and books (19%, 75/391) were the most popular restriction criteria reported. Specialists were more likely than general practitioners to restrict their search results to journals (50%, 130/258 vs 39%, 29/75; χ^2^_1_=5.951, *P*=.015) while general practitioners were more likely to restrict their search by source (45%, 34/75 vs 33%, 86/258; χ^2^_1_=4.911, *P*=.0026).

### How Much Time Do Physicians Spend on Searches?

The average amount of time that physicians were willing to devote to complex queries was between 10-20 minutes (Table 3). In comparison to other groups, medical professors and physicians in training devoted the largest amount of time to complex queries. General practitioners reported devoting the least amount of time to complex queries. A substantial proportion (46%, 209/453) of physicians reported frequently checking second and third pages of results.

Physicians in training devoted more time to complex queries than qualified physicians (Mode: 20-30 minutes vs 10-20 minutes; χ^2^_4_=9.619, *P*=.047). In total, 39% (21/54) of medical professors reported that they would be prepared to devote more than 30 minutes to a complex query. Medical professors were more likely to devote time to complex queries (χ^2^_4_=25.3028, *P*<.001) and check the second and third page of search results (χ^2^_2_=10.9823, *P*=.004) than specialists without professorship. Thus, it appears that they had more time to search more comprehensively.

### How Successful Are Physicians at Searching the Web?

Table 4 illustrates how often physicians fail at retrieving information from the Internet, why they think they failed, and what they usually did if they could not find the required information.

Overall, 59% (266/453) of physicians reported “sometimes”, “often”, or “always” being confronted with situations where they fail to retrieve the medical information they require from the Internet. As many as 15% (67/453) reported that this happened on a frequent basis (“often”, “always”).

When asked about situations where answers to medical questions could not be found on the Internet, most participants (76%, 278/368) indicated that an excess number of search results made it too time consuming to select relevant information, and 24% (90/368) were not sure how to formulate their query. Qualified physicians were more likely (27%, 81/298 vs 10%, 4/41; χ^2^_1_=6.121, *P*=.013) to express difficulties in formulating search queries than physicians in training.

As a consequence of failing to find the answer to a medical question, most participants (61%, 261/427) reported doing another, more specific search, and 8% (34/427) reported consulting a colleague when failing to find the answer on the Internet.

### What Are the Barriers to Finding Information?

Inaccessibility to relevant information, lack of time, and questionable trustworthiness were the most prominent barriers mentioned (Table 2 in Multimedia Appendix 2). Other important barriers were the absence of good quality filters/ratings as well as the questionable trustworthiness of search results.

In line with the findings illustrated in Table 3, general practitioners were more likely than specialists to perceive a lack of time to find relevant information as a barrier to obtaining medical information online (59%, 50/85 vs 43%, 111/260; χ^2^_1_=7.231, *P*=.007). Physicians in training were more likely than qualified physicians to perceive inaccessibility of relevant information (χ^2^_1_=6.7742, *P*=.009), too general search results (χ^2^_1_=4.884, *P*=.0027), and questionable trustworthiness (χ^2^_1_=8.045, *P*=<.001) as posing problems within the medical search domain. The finding that “search results appeared too general” is not surprising, since, as noted earlier, most participants reported the frequent reliance on generic search engines and social media when obtaining medical information.

**Table 3:** Time that physicians report having for answering complex questions (question: “How much time can you or are you generally willing to spend on trying to find the answer to an important, complex clinical question?”).

	Level of qualification		Level of medical specialization		Level of academic specialization		
	Qualified Physician % (n)	Physician in Training % (n)	General practitioner % (n)	Specialist % (n)	Specialist without professorship % (n)	Medical professor % (n)	Overall % (n)
< 10 min.	14 (50)	6 (3)	19 (17)	12 (33)	14 (30)	6 (3)	13 (53)
10-20 min.	33 (122)	28 (15)	38 (34)	32 (88)	33 (73)	28 (15)	33 (137)
20-30 min.	23 (85)	32 (17)	17 (15)	25 (70)	25 (55)	28 (15)	24 (102)
30-40 min.	17 (62)	11 (6)	18 (16)	17 (46)	19 (43)	6 (3)	16 (68)
> 40 min.	12 (46)	24 (13)	8 (7)	14 (39)	10 (21)	33 (18)	14 (59)
Total	100 (365)	100 (54)	100 (89)	100 (276)	100 (222)	100 (54)	100 (419)
Central tendency	Mode: 10-20 minutes	Mode: 20-30 minutes	Mode:	Mode: 10-20 minutes	Mode: 10-20 minutes	Mode: More than 40 minutes	Mode: 10-20 minutes
Statistical significance	χ^2^_4_=9.619, *P*=.047		Not significant		χ^2^_4_=25.3028, *P*<.001		

**Table 4:** Overall frequency, reason, and consequence of failure to retrieve medical information from the Internet.

Question	Categories	% (n/N) ^a^
**Frequency of failure: “How often do you face the situation where you cannot find the answer to a medical question on the Internet?**		
	Never, Rarely	41 (187/453)
	Sometimes	44 (199/453)
	Often, Always	15 (67/453)
**Reason of failure: “What is the most common reason you failed to find an answer?”**		
	Too many search results, too time-consuming to choose	76 (278/368)
	I was not sure how to formulate the query	24 (90/368)
**Consequence of failure**		
	Do another search using search terms that get MORE SPECIFIC	61 (261/427)
	Do another search using search terms that get LESS SPECIFIC	19 (81/427)
	Nothing, I stop searching on the Internet	9 (40/427)
	I send an email/Skype/chat with a colleague	8 (34/427)
	I post the question in a medical forum/physician community	3 (11/427)

^**a**^N is based on the number of respondents that provided a response to the corresponding question in the questionnaire.

### Search Tools and Advanced Search Options

**Figure 2:**
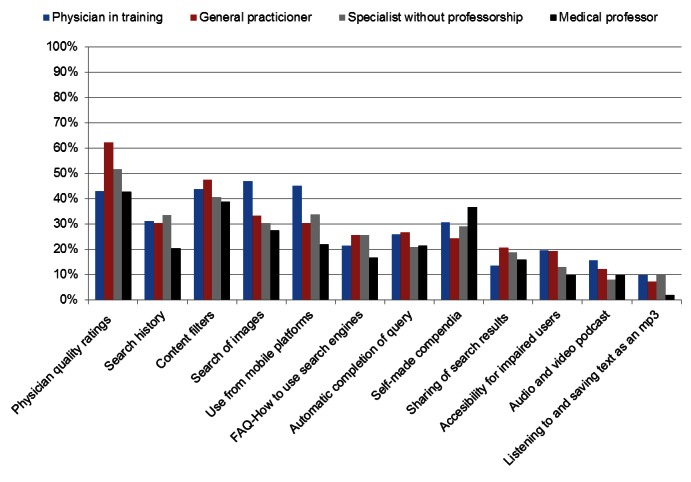
Responses to the question "How important do you perceive the following tools?", percentages of responses with the answer "Important" are illustrated.

As shown in Figure 2 and Table 3 in Multimedia Appendix 2, the search engine features physicians reported to be most important or desired were the possibility of “being able to quality rate medical information and perceive ratings of other physicians” (52%, 212/410), advanced search (45%, 188/422), being presented a list of popular websites (45%, 181/403), suggested relevant topics (39%, 159/413), search of images (33%, 133/409), and use from mobile platforms (33%, 137/412). The 15 most important tools are illustrated. Numerical values and statistical parameters are shown in Table 3 in Multimedia Appendix 2.

Physicians in training placed substantial importance on a wide variety of search tools, especially collaborative tools (physician quality ratings), media tools (mobile accessibility, image search), and data selection tools (advanced search). Congruent with these findings, it was found that physicians in training were more likely to report using mobile devices (*P*<.01) and placed higher importance on the use of video for the presentation of medical information (*P*=.022) than qualified physicians. Qualified physicians were more likely than physicians in training to place importance on the possibility to integrate patient data in the search process (36%, 124/343 vs 18%, 9/50; χ^2^_2_=6.617, *P*=.037). On the other hand, physicians in training were more likely than qualified physicians to regard the existence of a spelling correction tool as important (31%, 16/51 vs 18%, 61/348; χ^2^_2_=5.929, *P*=.052). A possible explanation for this finding is that it could be assumed that they have less experience in using medical terminology.

General practitioners were more likely than specialists to perceive the availability of medical calculators as important (42%, 34/82 vs 30%, 77/258; χ^2^_2_=6.538, *P*=.0038) and primarily sought collaborative and data selection tools. Furthermore, they were more likely than specialists to use the advanced search options of restricting results by language (48%, 43/89 vs 35%, 97/278; χ^2^_1_=5.148, *P*=.023*) and country (23%, 20/89 vs 13%, 36/278*; χ^2^_1_=4.728, *P*=.003). Some of this effect could be attributed to the fact that general practitioners reported lower levels of medical English competence than specialists.

Being able to restrict results by date (45%, 190/422) and language (36%, 153/422) were popular features, while filtering search results by format (13%, 56/422) was comparatively uncommon. Of the physicians whose mother tongue was not English, 26% (145/391) reported using language filters. Although language was not explicitly reported as a frequent barrier and levels of medical English competency were reported as high, a substantial proportion of physicians appear to prefer content being presented in their mother tongue. Furthermore, moderate importance was assigned to language tools such as automatic completion of queries (23%, 95/409), spelling correction (19%, 78/415), and automatic translation (15%, 65/421).

## Discussion

### Principal Findings

In line with previous research [6], general-purpose search engines (eg, Google), medical research databases (eg, PubMed), and Wikipedia were popular resources, while specialized search engines were unpopular. The popularity of medical society websites (ie, websites representing organized groups of physicians) was somewhat surprising. This could be explained by the fact that these websites often contain large amounts of peer-reviewed articles and medical news.

General practitioners were primarily interested in secondary resources including general, “easy to use” health content and collaborative resources (eg, physician network communities, forums). In contrast, specialists, especially medical professors, expected to access primary resources (eg, scientific journals, PubMed abstracts). Qualified physicians liked to access medical society websites while physicians in training were drawn towards encyclopedic resources such as Wikipedia and reported consulting websites suggested by their colleagues.

Current medical search engines are often based solely around primary resources and may consequently fail to address the resource expectations of a substantial fraction of physicians. Even though point-of-care databases (eg, UpToDate) provide reliable, evidence-based clinical information, the reported use among physicians was shown to be limited. A possible explanation could be that most physicians are not willing to pay high subscription fees to access medical information. In line with this notion, our study revealed that more than half of the physicians in our sample prefer advertisement-driven, free search services to paid services.

Our findings suggest that all groups of physicians, except general practitioners, are prepared to devote a considerable amount of time to important complex queries. However, physicians often fail to find the required information online. Known barriers to medical information retrieval such as inaccessibility of relevant information, questionable trustworthiness, and information overload [12] are confirmed by our findings. However, it appears that physicians either lack the time (general practitioner) or the skill (physician in training) to perform adequate data selection and evaluation when confronted with vast amounts of information.

The popularity of data selection tools (eg, suggested relevant topics, popular websites where most users found the answer) supports the notion that physicians seek help in finding relevant search results. The importance of the medical colleague in answering clinical questions has been verified over the last decade [6,17]. However, it has remained unclear to what extent the opinion of an anonymous colleague on the Internet can provide the needed “affirmation and support” [2]. Some support for the importance of “digital colleagues” was provided by the substantial fraction of physicians requesting collaborative tools (eg, functionality allowing them to share and perceive physician quality ratings of medical content). A general practitioner might, for example, seek the feedback of a specialist to obtain help in verifying content from general health websites. A specialist may seek experienced digital colleague support to obtain help in answering a complicated specific medical question. Thus, our data suggest that physicians seek online colleague support and feedback to aid them in data selection and medical decision making.

The Internet provides physicians with the opportunity to actively communicate with colleagues all over the world and to share information in the context of open-access platforms. Our results confirm previous findings suggesting the growing dominance of social media in the medical health domain [18,19]. Social media have previously been defined to include both “professional physician platforms” as well as encyclopedic open-access resources such as Wikipedia [19]. While creation and usage of a specialized medical Wikipedia appears like an interesting solution [20], the success of such projects has been mixed [19]. A differentiated approach may be necessary. We found that physicians in training are the most likely subgroup to use Wikipedia while general practitioners are most likely to use physician communities.

Medical professors reported the least reliance on collaborative and data selection tools and primarily used tools such as self-stored compendia, which can aid the preparation of presentations or manuscripts. A possible explanation could be that medical professors are more proficient in online data selection due to high levels of research experience and consequently require less help from other physicians.

An interesting finding was that despite language not being mentioned as an explicit search barrier, and self-perceived understanding of medical English being high, many physicians, especially general practitioners, reported restricting their search results by language. Thus, it appears that many physicians residing outside the English-speaking domain are interested in local information in their mother tongue. Possible explanations could be a preference to read their mother tongue and increased relevance of local resources to their health care settings.

### Strengths and Weaknesses of the Study

At the time of this writing and to our best knowledge, the current study is based on the largest sample size of all detailed European scientific studies on the professional use of the Internet by medical practitioners. With regard to the large number of questions answered by each participant, it is one of the most extensive studies in the field. A potential weakness of the study is that it is biased against medical practitioners who use the Internet rarely, since the questionnaires were primarily disseminated via email, and the online promotion and the questionnaire were available only through a website. Another potential weakness is that the study is solely based on self-report, while physicians have been found to overreport their use of objective resources and underreport their reliance on subjective sources such as colleagues [17]. Third, physicians from certain European countries (Austria, Switzerland, United Kingdom, and France) and physicians living in urban areas are overrepresented in the sample.

### Possible Explanations and Implications for Clinicians and Policy Makers

General search engines are popular but offer vast amounts of often irrelevant, invalid information that require physicians to pursue substantial data screening and filtering. Appropriate tools may help to overcome this problem. In terms of usability, simple search systems such as Wikipedia and Google appear to attract physicians, possibly due to time constraints but also due to their level of information need. A substantial proportion of physicians seek secondary resources, struggle with limited access to readable, high-quality content, and lack the time and skill to pursue relevant data selection. Since the quality of medical information accessed on the Internet is likely to increasingly impact medical care, it may be of importance that all physicians are trained in efficient online data selection and have easy accessibility to high-quality resources. A potential solution might also be governmental support of the development of openly available, effective medical search engines, open access to high-quality content for physicians and the improvement of the quality of popular existing Web resources such as Wikipedia.

### Unanswered Questions and Future Research

Our research provides the investigative basis for questions that need to be answered in further experimental research. The finding that most clinicians use Wikipedia and general search engines to obtain medical information raises the question of the impact of such behavior on the quality of medical care. It also needs to be investigated why physicians are largely unwilling to pay for high-quality medical information services, and what alternative financing models for such services could look like. It is unknown to what extent different types of physicians have the expertise to evaluate the reliability of content provided on the Web. Level of initial knowledge or expertise may play a central role in determining data selection skills and the type of resources expected when searching for information. The extent to which initial knowledge of a medical topic has an impact on the type of medical resource used could be clarified in experimental research. Further quantitative research representing different health systems, including a higher proportion of younger physicians and comparing different areas of medical specialization, is needed.

## Multimedia Appendix 1

Physician questionnaire (English).

## Multimedia Appendix 2

Tables.
