# Supplementary material for: Utilization and Perceived Problems of Online Medical Resources and Search Tools Among Different Groups of European Physicians
Source: J Med Internet Res. 2013 Jun 26;15(6):e122. doi: 10.2196/jmir.2436 (PMC3713956; doi:10.2196/jmir.2436)
Supplement: Supplementary file 1 [file jmir_v15i6e122_app1.pdf]

Are you a physician or final year medical student?

1) **Yes**
2) **No**

Button "Proceed"

If yes – redirected to the questionnaire

If no – "Thank you for your interest in our study, however, it is addressed to the medical doctors and medical students of last years of studies. If, you want to keep in touch with us and participate in other studies, please leave your email: (blank field for the email)"

How do you search for medical information on the Internet?

The following survey has been designed for medical doctors (in training or qualified) of all specialties.

The questionnaire was developed by the **Society of Physicians in Vienna** (link to www.billrothhaus.at) in collaboration with the Health on the Net Foundation, within the ramework of the European Project KHRESMOI (2010-2014).

Your participation will enable a better understanding on how medical doctors search for online medical information and help identify problems with current solutions. The results of the survey will contribute to the creation of a new medical search engine, adjusted to the requirements of medical doctors.

The survey takes about 20 minutes to complete.

All information collected is used exclusively for the purpose of the study and will be kept confidential. We do not collect personally identifiable information without your consent. More information on the Confidentiality and data privacy usage.(link to Confidentiality page)

There will be free online access to results after the completion of the survey.

Contributions from around the world are welcome.

Part 1: Internet Access

1.1 How long have you been using the Internet?

1 Less than 1 year

2 1-5 years

3 5-9 years

4 10-14 years

5 15 years or more

1.2 Do you currently have regular Internet access?

1 Yes

2 No

1.3 How often do you connect to the Internet?

1 Daily

2 Few times a week

3 Once a week

4 Few times a month

5 Once a month or less

1.4 How do you connect to the Internet?

(Multiple Answers possible)

1 Through a modem / cable or DSL

2 Through a Wi-Fi connection

3 Through a USB modem

4 Through a mobile phone

5 Other please, specify

6 Do not know

1.5 Which device(s) do you use to connect to Internet?

(Multiple Answers possible)

1 PC at home or in office

2 Laptop/Net book

3 Mobile phone

4 Smartphone (e.g. iPhone, Blackberry, Asus, LG etc.)

5 Tablets (iPad, Samsung Galaxy, Android 2.1 Tablet PC, Fly touch 1 , View sonic View pad etc.

6 Other :________(Please specify)

1.6 How often do you access the Internet during a medical visit/ patient consultation?

1 Never

2 Rarely

3 Sometimes

4 Often

5 Always

6 I never see patients

1.7 What is the device you use <u>the most</u> to access <u>medical information/do medical updating</u> on the Internet, in the following situations?

	PC	Laptop, Net book	Mobile phone	Smartphone (e.g. iPhone, Blackberry, Asus etc.)	Tablet (e.g. iPad, Samsung Galaxy etc.)	I never access medical information/do medical updating in this situation	Other: (Please specify
During a consultation with a patient							
At work without a patient							
During a colloquium or meeting							
During a conference and while travelling							
At home							

1.8 Do you access or take part in online physician network communities? (e.g. doc2doc, medting, medext)

1 **Yes, I access_______**(Please specify)

2 **No**

3 **I have never heard of such communities**

Part 2 Medical Information Need

2.1 What kind of medical information do you look for as part of your daily practice?

Answering possibilities: Never, Rarely, Sometimes, Often, Always

1 Abbreviations/Definitions

2 Diagnostic aid

3 Disease descriptions

4 Treatment specific information

6 Drug information

7 Prevention

9 Causes and risk factors

10 Clinical trial information/scientific literature

11 Patient resources: Information/leaflets for patients (e.g. support groups)

12 Job/Career opportunities

13 Medical equipment

14 Information about other institutions /doctors for referral

15 Medical education/Conferences

16 Other :_______(Please specify)

2.2 What kind of drug related information do you look for as part of your daily practice?

Multiple Answers possible

1 Drug description

2 Drug side effects

3 Drug interactions

4 Contraindications

5 Drug safety, toxicity

6 Drug dosage

7 Drug prices

9 New medication/Medication in development

10 Other: ____________(Please specify)

2.3 When do you use the Internet <u>the most</u> for professional purposes?

1 When I require an immediate answer (i.e. within 10 minutes) Please, add examples:

2 When I require a non immediate answer (i.e. within 2 days) Please add examples:

3 To fulfill my educational needs (e.g. CME)

Part 3   Information Resources

3.1 How often do you use the following types of online sources to find online medical information? Answering possibilities: Never, Rarely, Sometimes, Often, Always

1 General search engines (Google, Yahoo, MSN etc.)

2 Wikipedia

3 General health related websites

4 Pharmaceutical company websites

5 Hospital or University websites

6 Medical research databases (e.g. Pub med, Cochrane Library)

7. Specialized medical search tool (e.g. HON Select, Yottalook)

8. Point-of-Care/evidence-based medical databases (e.g. UpToDate, Best Practice)

9. Targeted/area specialized websites

10. Society websites (e.g. Society of Physicians, Medical Council)

11. Web sites suggested by a colleague

12. Physician network communities

13. Medical forums/Blogs

14. Other________(Please specify)

3.2 How often do you usually carry out the following actions as a result of obtaining clinical information online? Answering possibilities: Never, Rarely, Sometimes, Often, Always

1 Change a patient's medication

2 Modify a patient's treatment/therapy

3 Print out information for the patient or recommend a website to the patient

4 Recommend to a patient a behavior change of habits (e.g. lifestyle)

5 Recommend further tests based on symptoms

6 Ask a colleague for his/her opinion

7 Request more information about a product or medication

8 Conduct further research using other resources

9 Other :_____(Please specify)

Part 4 Search Behavior

4.1 How much time can you/are you willing to spend on trying to find the answer to an important complex clinical question? (Answer required within two days)

1 Less than 10 min

2 10-20 minutes

3 20-30 minutes

4 30-40 minutes

5 More than 40 minutes

4.2 How do you usually phrase your search when using an online search engine?

1 In question format (i.e. "What is the link between X and Y?")

2 Using single key words (i.e. " X, Y, Link)

3 Other please, specify ____________

4.3 What do you usually first type into the search bar when you want to find out more about a medical problem?

1 The Diagnosis (e.g. heart disease)

2 The Symptom (e.g. chest pain)

4 Other ________ (Please, specify)

4.4 Which advanced search options do you mainly use when searching for medical information?

Multiple answers possible

1 Date range

2 Country

3 Language

4 Document format (pdf, doc, ppt)

5 Other please, specify

6 I do not use advanced search options

4.5 **Which restriction criteria do you use when searching for medical information?**

Multiple Answers possible

1 Image

2 Video

3 Type of Source (e.g. medical professional/layperson, University/private institution)

4 News

5 Books

6 Journals

7 Automatic Translation of results

8 Blogs

9 Other:______(Please specify)

10 I do not use restriction criteria

4.6 **When search results (list of links) appear, where do you usually click first?**

1 On the first link

2 On the link which appears to have the most trustworthy source

3 On the link which looks most relevant

4 Other: ____________(Please specify)

4.7 **How often do you check the second or third page of results?**

1 Never

2 Rarely

3 Sometimes

4 Often

5 Always

4.8 How often do you use the following criteria to rate the quality of the information you retrieve from the Internet?

Answering possibilities: Never, Rarely, Sometimes, Often, Always

1 Author (e.g. medical professional vs. lay person)

2 Publisher/Institution (University vs. health organization, Thieme vs. Elsevier)

3 Time of last update

4 References to the original source

4.9 How often do you face situations where you cannot find the answer to a medical question in the Internet?

1 Never

2 Rarely

3 Sometimes

4 Often

5 Always

4.10 What is the most common reason you failed to find an answer?

1 Too many search results, too difficult /time-consuming to choose what is relevant

2 I was not sure how to formulate the search query

3 Other __Please, specify_____________

4.11 What do you usually do when you search for a medical problem on the Internet and the relevant answer does not show up? Multiple Answers possible

1 Do another search using search terms that get MORE SPECIFIC

2 Do another search using search terms that get LESS SPECIFIC

3 Nothing, I stop searching on the Internet

4 I send an E-mail/Skype/Chat with a colleague

5 I post the question in a medical forum/physician community website

6 Other________(Please, specify)

Part 5 Barriers

5.1 What difficulties do you face when searching for medical, health and prescription information on the internet?

Multiple Answers possible

1 **Slow internet connection**

3 **Time consuming to find relevant information**

4 **Search results are too general**

5 **Search results are too specific**

6 **Questionable trustworthiness**

7 **“Restricted accessibility” to good quality information (e.g. membership or subscription required)**

8 **Absence of good quality filter/rating**

9 **Lack of Usability for smart phones and mobile devices**

10 **Distracting advertisements**

11 **Language Barrier: Insufficient medical information available in my mother tongue**

12 **Other ____________(Please specify)**

Part 6 Expectation/Ideal Search Engine

6.1 How important are the following characteristics of a search engine to you?

Answering possibilities: Unimportant, of little importance, moderately important, Important, very important

1 Speed

2 Relevance of results

3 Trustworthiness of search results

4 Readability of results

5 Quality of link description

6 Possibility to view search history

7 Easy-to-use / simplicity

8 Usability for smart phones and mobile devices

9 Advanced Options/Filters

9 Other:__________(Please specify)

6.2 How many search results do you prefer to be displayed on a page?

1 No more than five

2 Between five and ten

3 Between ten and twenty

4 More than twenty

6.3 Please, choose how you prefer the search results to be categorized?

Multiple answers possible

1 Type of author (health professional / non health professional)

2 Type of target audience (general public, patients, and health professionals)

3 Type of content (articles vs. blogs and forums)

4 Quality accreditation marks (HONcode, URAC, WMA etc)

5 Type of organization (Hospital, Pharmaceutical company, University, Insurance company, Government)

6 Other:______(Please specify)

6.4 How important do you rate the following media formats for the presentation of medical information? Answering possibilities: Unimportant, of little importance, moderately important, Important, Very important

1 Video

2 Audio

3 Text and Graphic (Image, Picture)

6.5 How important do you rate the following tools?

Answering possibilities: Unimportant, Neutral, Important

1 Advanced search (country, language, date range)

2 Automatic translation

3 Suggested relevant topics

4 Spelling correction

5 Automatic completion of query

6 Search of images

7 FAQ -how to use the search engine

8 Word cloud representing the most prevalent words across the search results by a search engine

9 Sharing your search results with your colleague, patient or friend (by email)

10 Receiving updates of your search results by email/RSS

11 Ability to listen to the text and save it as mp3

12 Accessibility for impaired users

13 Audio and video podcast

14 Use from mobile platforms

15 Possibility to view search history

16 Medical calculators

17 Being able to quality rate information/websites and perceiving the ratings of other physicians

18 Integration of patient data within the search process as a diagnostic aid for complex cases

19 Possibility to store self-made compendiums

20 Possibility to choose between content filters (disease description, drugs information)

21 A list of “popular” websites (i.e. where most users have found the answer to medical query)

22 Other:_____ (Please specify)

6.6 Would you prefer….?

1 To have ads in order to keep a search service free

2 To donate and avoid advertisement placement

3 To pay fee-for-service each time you use the search engine (Please, indicate how much per query (Euro))

4 To subscribe to a membership subscription (Please, indicate how much per month (Euro)

6.7 Please describe any other ideas/suggestions towards an ideal medical search engine or tool helping you find medical information on the internet.

Part 7 Tell us about yourself

7.1 Your age for all

1 20-29

2 30-39

3 40-49

54 50-59

5 60-69

6 70-79

7 >=80

7.2 Gender Male

Female

7.3 Where do you live and work?

1 I live and work in urban area

2 I live and work in rural area

3 I live in rural area and work in urban

4 I live in urban area and work in rural

7.4 What is your highest completed academic degree?

1 I am a medical student and have not completed my degree yet

2 Medical degree

2 Medical degree plus Master

3 Medical degree plus PhD

4 Medical degree plus Professorship/Habilitation

5 Other _____________please specify

7.5 Do you currently work as a physician?

1 Yes, I work as a qualified general practitioner

2 Yes, I work as a qualified specialist

2 Yes, but I am a specialist/general practitioner in training

3 No, I am currently unemployed / retired

4 No, I work in another field

5 Other :_____________(Please specify)

7.6 How long have you worked as a qualified (i.e. completed medical degree) physician?

1 Less than 12 months

2 1-3 years

3 4-6 years

4 7- 9 years

5 10-20 years

6 More than 20 years

7 I am a final year medical student

8 I have completed my medical degree, but have not worked as a physician

9 Other_____________________(Please specify)

7.7 Where do you work? (Please refer to your main job)?

1 I am self-employed

2 I work in a public healthcare service

3 I work in a private healthcare service

4 I work in a university medical healthcare service

5 I am unemployed

6 I am retired

7 Other

7.8 How many patients do you consult on average per (working) day?

1 I never consult patients

2 Below 20

3 20-40

4 41-60

4 More than 60

5 Other:______________(please specify)

7.9 What is your main specialization?

1) **Anesthesiology and Critical Care Medicine**

2) Dermatology

3) Emergency Medicine

4) General Practice

5) Child- and Adolescent Medicine

6) Internal Medicine

7) Pulmonary Disease

8) Neurology/Psychiatry

9) Obstetrics and Gynecology

10) Ophthalmology

11) Orthopedics and orthopedic. Surgery

12) Otolaryngology (Nose, Ear, Throat)

13) Physical Medicine and Rehabilitation

14) Surgery

15) Urology

16) Orthodontist and Dental Medicine

17) Radiology

18) Research and Education________(Please specify main field)

19) Others___________(Please specify)

20) I am a physician in training___________(Please specify field)

7.11 Country

1	Albania
2	Austria
3	Belgium
4	Bulgaria
5	Bosnia
6	Canada
7	China
8	Croatia
9	Cyprus
10	Czech Republic
11	Denmark
12	Estonia
13	Finland
14	France
15	Germany
16	Greece
17	Hungary
18	Iceland
19	India
20	Ireland
21	Israel
22	Italy
23	Kosovo
24	Latvia
25	Lichtenstein
26	Lithuania
27	Luxembourg

28	Macedonia
29	Malta
30	Montenegro
31	Netherlands
32	Norway
33	Poland
34	Portugal
35	Romania
36	Russia
37	Serbia
38	Slovakia
39	Slovenia
40	Spain
41	Sweden
42	Switzerland
43	The UK
44	The USA
45	Turkey
46	Other Please, specify

7.12 Mother tongue

1	Albanian
2	Arabic
3	Bosnian
4	Bulgarian
5	Chinese
6	Croatian
7	Czech

**8**	**Danish**
**9**	**Dutch**
**10**	**English**
**11**	**Estonian**
**12**	**Finnish**
**13**	**French**
**14**	**German**
**15**	**Greek**
**16**	**Hindi/Urdu**
**17**	**Hungarian**
**18**	**Icelandic**
**19**	**Irish**
**20**	**Italian**
**21**	**Japanese**
**22**	**Latvian**
**23**	**Lithuanian**
**24**	**Luxembourgish**
**25**	**Macedonian**
**26**	**Malay**
**27**	**Maltese**
**28**	**Norwegian**
**29**	**Polish**
**30**	**Portuguese**
**31**	**Romanian**
**32**	**Russian**
**33**	**Serbian**
**34**	**Slovakian**
**35**	**Slovene**
**36**	**Spanish**

37 Swahili

38 Swedish

39 Turkish

40 Other

7.13 How well do you understand medical English?

1 English is my mother tongue

2 Excellent

3 Very well

4 Well

5 Average

6 Poor

7 I don't understand any English

7.14 Please indicate in the following field the Homepage of your Internet browser (Internet Explorer, Firefox, Safari and Opera): ______________________________________

Please indicate the page in the format: www.homepage.com

If you don´t know your Homepage just, open your Internet Browser and copy and paste the first available site in this field:

7.15 The data you have provided is confidential. If you are willing to be contacted for follow up studies or would like to be informed of results please give us your E-Mail address:

1 Yes, my E-Mail address is_____________

2 No

THANK YOU FOR YOUR PARTICIPATION!
