# Supplementary material for: Utilization and Perceived Problems of Online Medical Resources and Search Tools Among Different Groups of European Physicians
Source: J Med Internet Res. 2013 Jun 26;15(6):e122. doi: 10.2196/jmir.2436 (PMC3713956; doi:10.2196/jmir.2436)
Supplement: Supplementary file 2 [file jmir_v15i6e122_app2.pdf]

Multimedia Appendix 2. Tables.

Table 1. Online resources used for obtaining medical information (the percentage of respondents per group that claimed to use a given resource “often” or “always” when searching for medical information on the Internet; χ^2 and *P* values are shown for comparisons in which statistically significant differences were identified).

	Level of qualification % (n/N)[a]		Level of medical specialization % (n/N)		Level of academic qualification % (n/N)	
	Qualified physician	Physician in training	General practitioner	Specialist	Specialist without professorship	Medical professor
General search engine	78 (287/367)	82 (45/55)	76 (68/89)	79 (219/278)	79 (177/244)	78 (42/54)
Medical research databases	58 (209/362)	69 (38/55)	27 (24/88)	68 (185/274)	64 (141/220)	82 (44/54)
			χ^2_2=44.905, *P*<.001		χ^2_2=7.461, *P*=.024	
Wikipedia	37 (131/353)	56 (31/55)	35 (30/86)	38 (101/267)	34 (74/215)	52 (27/52)
	χ^2_2=8.997, *P*=.011				χ^2_2=6.619, *P*=.037	
General society websites	41 (147/361)	29 (16/55)	34 (30/88)	43 (117/273)	43 (95/221)	42 (22/52)
	χ^2_2=11.622, *P*=.003					
Hospital/university websites	27 (95/357)	38 (21/55)	20 (17/86)	29 (78/271)	28 (61/219)	33 (17/52)
	χ^2_2=6.409, *P*=.041					
Targeted/area specialized websites	32 (114/354)	22 (12/55)	28 (24/87)	34 (90/267)	34 (74/216)	31 (16/51)
General health related websites	23 (82/356)	19 (10/54)	36 (31/87)	19 (51/269)	20 (43/218)	16 (8/51)
			χ^2_2=12.813, *P*=.002			
Websites suggested by a colleague	10 (37/355)	15 (8/54)	14 (12/84)	9 (25/271)	9 (20/219)	10 (5/52)
	χ^2_2=8.653, *P*=.001					
Point-of-care/evidence-based medical databases (eg, UpToDate)	25 (89/361)	27 (15/55)	26 (23/88)	24 (66/273)	24 (53/221)	25 (13/52)
Pharmaceutical websites	8 (27/357)	11 (6/55)	9 (8/86)	7 (19/271)	7 (15/219)	8 (4/52)
Physician network communities	9 (32/356)	6 (3/55)	17 (15/86)	6 (17/270)	6 (13/219)	8 (4/51)
			χ^2_2=9.841, *P*<.001			
Medical forums/blogs	6 (22/352)	2 (1/55)	12 (10/86)	5 (12/266)	4 (8/216)	8 (4/50)
			χ^2_2=9.841, *P*<.01			
Specialized medical search tool	6 (21/352)	4 (2/55)	7 (6/85)	6 (15/267)	7 (6/85)	2 (1/52)

[a] N is based on the number of respondents in a specific subgroup that provided a response to the corresponding question in the questionnaire.

Table 2. Reported barriers to finding medical information online (multiple answers were allowed to the question: “What difficulties do you have when looking for medical information online?”; the χ^2 and *P* values are shown for comparisons in which statistically significant differences were identified).

<table>
<tr><th></th><th colspan="2">Level of medical qualification</th><th colspan="2">Level of medical specialization</th><th colspan="2">Level of academic qualification</th><th>Overall</th></tr>
<tr><th></th><th>Qualified physician % (n)</th><th>Physician in training % (n)</th><th>General practitioner % (n)</th><th>Specialist % (n)</th><th>Specialist without professorship % (n)</th><th>Medical professor % (n)</th><th>% (n)</th></tr>
<tr><td></td><td></td><td></td><td></td><td></td><td></td><td></td><td></td></tr>
<tr><td rowspan="2">Restricted accessibility to good quality information</td><td>64 (220)</td><td>81 (43)</td><td rowspan="2">64 (54)</td><td rowspan="2">64 (166)</td><td rowspan="2">63 (133)</td><td rowspan="2">69 (33)</td><td rowspan="2">65 (279)</td></tr>
<tr><td colspan="2">χ^2_1=6.774, *P*<.001</td></tr>
<tr><td rowspan="2">Time consuming to find relevant information</td><td rowspan="2">47 (161)</td><td rowspan="2">38 (20)</td><td>59 (50)</td><td>43 (111)</td><td rowspan="2">45 (96)</td><td rowspan="2">31 (15)</td><td rowspan="2">45 (193)</td></tr>
<tr><td colspan="2">χ^2_1=7.231, *P*=.007</td></tr>
<tr><td rowspan="2">Questionable trustworthiness</td><td>30 (104)</td><td>49 (26)</td><td rowspan="2">31 (26)</td><td rowspan="2">30 (78)</td><td rowspan="2">30 (63)</td><td rowspan="2">31 (15)</td><td rowspan="2">33 (143)</td></tr>
<tr><td colspan="2">χ^2_1=8.045, *P*<.001</td></tr>
<tr><td rowspan="2">Search results are too general</td><td>31 (106)</td><td>45 (24)</td><td rowspan="2">31 (26)</td><td rowspan="2">31 (80)</td><td rowspan="2">33 (69)</td><td rowspan="2">23 (11)</td><td rowspan="2">33 (141)</td></tr>
<tr><td colspan="2">χ^2_1=4.884, *P*=.027</td></tr>
<tr><td>Absence of good quality filter/rating</td><td>31 (107)</td><td>26 (14)</td><td>33 (28)</td><td>30 (79)</td><td>32 (67)</td><td>25 (12)</td><td>30 (128)</td></tr>
<tr><td>Distracting advertisement</td><td>23 (78)</td><td>26 (14)</td><td>18 (15)</td><td>24 (63)</td><td>22 (47)</td><td>33 (16)</td><td>24 (102)</td></tr>
<tr><td>Slow Internet Connection</td><td>16 (55)</td><td>18 (9)</td><td>19 (16)</td><td>15 (39)</td><td>14 (30)</td><td>19 (9)</td><td>16 (69)</td></tr>
<tr><td>Lack of usability for mobile devices (eg, smartphone)</td><td>10 (36)</td><td>17 (9)</td><td>15 (13)</td><td>9 (23)</td><td>8 (16)</td><td>15 (7)</td><td>12 (52)</td></tr>
<tr><td>Language barrier</td><td>5 (17)</td><td>4 (2)</td><td>8 (7)</td><td>4 (10)</td><td>3 (7)</td><td>6 (3)</td><td>5 (21)</td></tr>
<tr><td>Search results are too specific</td><td>4 (14)</td><td>11 (6)</td><td>4 (3)</td><td>4 (11)</td><td>4 (8)</td><td>6 (3)</td><td>5 (20)</td></tr>
<tr><td>Total</td><td>100 (345)</td><td>100 (53)</td><td>100 (85)</td><td>100 (260)</td><td>100 (212)</td><td>100 (48)</td><td>100 (430)</td></tr>
</table>

Table 3. Responses to the question "How important do you perceive the following tools?", percentages of responses with the answer "Important" are illustrated (the χ² and *P* values are shown for comparisons in which statistically significant differences were identified).

	Level of qualification % (n/N) [a]		Level of medical specialization % (n/N)		Level of academic qualification % (n/N)	
	Qualified physician	Physician in training	General practitioner	Specialist	Qualified specialist	Medical professor
Physician quality ratings	53 (182/344)	43 (22/51)	62 (51/82)	50 (131/262)	52 (110/213)	43 (21/49)
Advanced Search	43 (153/356)	51 (26/51)	41 (35/86)	44 (118/270)	47 (103/219)	29 (15/51)
					χ^2_2=11.066, *P*=.004	
A list of popular websites (where most users have found answers to queries)	46 (155/341)	46 (22/48)	53 (44/83)	43 (111/258)	48 (101/211)	21 (10/47)
					χ^2_2=11.192, *P*=.004	
Possibility to choose between content filters	42 (144/342)	44 (22/50)	48 (40/84)	40(104/258)	41 (85/209)	39 (19/49)
Suggested relevant links	41 (141/347)	26 (13/51)	46 (37/80)	39(104/267)	42 (90/216)	28 (14/51)
					χ^2_2=16.579, *P*<.001	
Search of images	31 (106/345)	47 (23/49)	33 (27/81)	30 (79/264)	31 (65/213)	28 (14/51)
Medical calculators	33 (111/340)	40 (20/50)	42 (34/82)	30 (77/258)	30 (63/208)	28 (14/50)
			χ^2_2=6.538, *P*=.0038			
Integration of patient data within search process as a diagnostic aid for complex cases	36 (124/343)	18 (9/50)	42 (34/81)	34 (90/262)	36 (76/212)	28 (14/50)
	χ^2_2=6.617, *P*=.037					
Possibility to view search history	31 (107/345)	31 (16/51)	31 (25/82)	31 (82/263)	34 (72/214)	20 (10/49)
Use from mobile platforms	31 (108/345)	45 (23/51)	31 (25/82)	32 (83/263)	34 (72/213)	22 (11/50)
FAQ, How to use search engines	25 (83/339)	22 (11/51)	26 (21/82)	24 (62/257)	26 (54/209)	17 (8/48)
Automatic completion of query	22 (77/344)	26 (13/50)	27 (22/82)	21 (55/262)	21 (44/211)	22 (11/51)
Possibility to store self-made compendia	29 (98/336)	31 (15/49)	24 (20/82)	31 (78/254)	29 (60/205)	37 (18/49)
Word cloud	23 (80/343)	24 (12/50)	23 (19/82)	23 (61/261)	25 (52/211)	18 (9/50)
					χ^2_2=6.280, *P*=.043	
Spelling correction	18 (61/348)	31 (16/51)	19 (15/81)	17 (46/267)	17 (37/217)	18 (9/50)
	χ^2_2=5.929, *P*=.052					
Sharing your search results with your colleague, patient of friend	19 (65/345)	14 (7/51)	21 (7/82)	18 (48/263)	19 (40/213)	16 (8/50)
Receiving updates of your search results by email/RSS	19 (65/342)	14 (7/50)	18 (15/82)	19 (50/260)	21 (45/210)	10 (5/50)
					χ^2_2=7.514, *P*=.023	
Automatic translation	16 (55/353)	9 (5/53)	20 (17/85)	14 (38/268)	16 (35/217)	6 (3/51)
					χ^2_2=9.429, *P*=.023	
Accessibility for impaired users	14 (49/345)	20 (10/51)	20 (16/82)	13 (33/263)	13 (28/213)	10 (5/50)
Audio and video podcast	9 (32/341)	16 (8/51)	12 (10/82)	9 (22/259)	8 (17/209)	10 (2/50)

Ability to listen to the text and save it as MP3	8 (28/341)	10 (5/51)	7 (6/82)	9 (22/259)	10 (21/209)	2 (1/50)

[a] N is based on the number of respondents that provided a response to the corresponding question in the questionnaire.
